# Combined Use of Cyclodextrins and Amino Acids for the Development of Cefixime Oral Solutions for Pediatric Use

**DOI:** 10.3390/pharmaceutics13111923

**Published:** 2021-11-13

**Authors:** Marzia Cirri, Natascia Mennini, Giulia Nerli, Jessica Rubia, Enrico Casalone, Fabrizio Melani, Francesca Maestrelli, Paola Mura

**Affiliations:** 1 Department of Chemistry, University of Florence, Via Ugo Schiff 6, 50019 Sesto Fiorentino, Italy; natascia.mennini@unifi.it (N.M.); giulia.nerli@unifi.it (G.N.); jessica.rubia050393@gmail.com (J.R.); francesca.maestrelli@unifi.it (F.M.); paola.mura@unifi.it (P.M.); 2 Department of Biology, University of Florence, Via Madonna del Piano 6, 50019 Sesto Fiorentino, Italy; enrico.casalone@unifi.it; 3 Department of Neuroscience, Psychology, Drug Research and Child Health, Section of Pharmaceutical and Nutraceutical Sciences, University of Florence, Via U. Schiff 6, 50019 Sesto Fiorentino, Italy; fabrizio.melani@unifi.it

**Keywords:** cefixime, amino acids, pediatric oral formulation, sulfobutylether-β-cyclodextrin, histidine, molecular dynamic studies

## Abstract

Cefixime (CEF) is a cephalosporin included in the WHO Model List of Essential Medicines for Children. Liquid formulations are considered the best choice for pediatric use, due to their great ease of administration and dose-adaptability. Owing to its very low aqueous solubility and poor stability, CEF is only available as a powder for oral suspensions, which can lead to reduced compliance by children, due to its unpleasant texture and taste, and possible non-homogeneous dosage. The aim of this work was to develop an oral pediatric CEF solution endowed with good palatability, exploiting the solubilizing and taste-masking properties of cyclodextrins (CDs), joined to the use of amino acids as an auxiliary third component. Solubility studies indicated sulfobutylether-β-cyclodextrin (SBEβCD) and Histidine (His) as the most effective CD and amino acid, respectively, even though no synergistic effect on drug solubility improvement by their combined use was found. Molecular Dynamic and ^1^H-NMR studies provided insight into the interactions of binary CEF:His and ternary CEF:His:SBEβCD systems used to prepare CEF solutions, which resulted stable and maintained unchanged antimicrobial activity during the two-weeks-use in therapy. The ternary solution was superior in terms of more tolerable pH (5.6 vs. 4.7) and better palatability, being resulted completely odorless by a panel test.

## 1. Introduction

**Figure 1:**
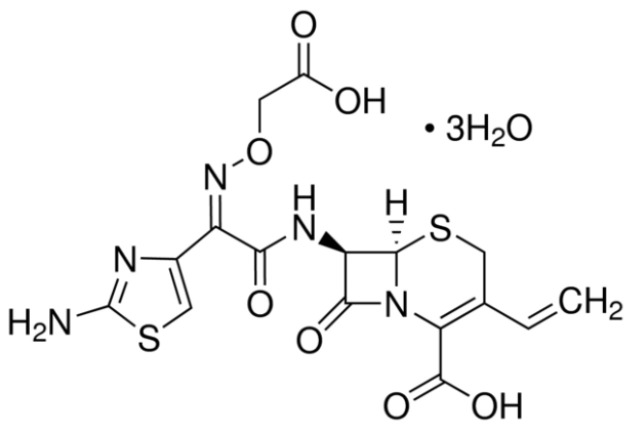
Chemical structure of CEF.

Cefixime (CEF) (Figure 1) is a third-generation cephalosporin considered essential for children up to 12-years old, being included in the WHO Model List of Essential Medicines for Children among the ‘Watch group antibiotics’ and recommended as essential second choice treatment for gastroenteritis and colitis without specification of infectious agent [1]. CEF is also active against β-lactamase-producing bacterial strains that provoke respiratory tract infections (e.g., acute bronchitis, pharyngitis and tonsillitis), otitis media, uncomplicated urinary tract infections and gonorrhea [2].

In the European market, CEF-based formulations are present as powders for oral suspensions (100 mg/5 mL) and tablets (200 and 400 mg). In order to meet the needs of the pediatric population and obtain acceptable formulation in the appropriate dose, dosage forms intended for adults such as tablets, capsules or concentrated suspensions are usually extemporaneously manipulated by crashing, opening or diluting, respectively. However, these operations can be harmful in pediatric patients: in fact, particularly when very low dosages are needed, a poor or uncontrolled dosing accuracy is the most frequent medication mistake [3].

Moreover, the impact of the above manipulations on the drug bioavailability is often unknown, and these extemporaneous formulations can have limited and unknown physical, chemical and microbiological stability, leading to enhanced risks of medication errors, and the appearance of adverse reactions [4,5,6,7].

Furthermore, extemporaneous preparations often have poor palatability and/or unpleasant taste that can heavily reduce the young patient’s compliance, and, consequently, the effectiveness of the therapy [8]. Liquid formulations are considered the best choice for pediatric use, as also reported by European Medicinal Agency (EMA), mainly due to their ease of dose- and weight-adaptability [9]. Moreover, liquid formulations are easier to swallow, thus improving the young patient’s compliance. Among liquid formulations, solutions are preferable to suspensions, due to the absence of the sediment that can produce choking risks, dosing errors and problems in enteral feeding administration. Oral solutions of drugs such as amlodipine [10,11,12] have been recently successfully developed, adapting the formulation to the pediatric use of these drugs.

The very low aqueous solubility of CEF contributes to its low absorption (40–50%) from the gastrointestinal tract [13] and makes it difficult the development of liquid formulations. Several efforts have been performed to overcome this important drawback, including preparation of CEF amorphous nanoparticles [14,15] or cocrystals with different coformers [16], as well as the use of solid dispersions with different hydrophilic carriers [17,18,19] or of cyclodextrins, alone or in combination with other solubilizing agents [20,21].

Cyclodextrins (CDs) are cyclic oligosaccharides with a hydrophilic outer surface and a lipophilic central cavity. Due to the hydrophobic properties of their interiors, CDs can form inclusion complexes in aqueous solvent with several molecules, which has been widely exploited in the pharmaceutical field for various purposes, such as solubility enhancement, chemical or physical stability improvement and taste-masking of drugs [12,22,23,24,25].

Orally disintegrating tablets (ODT) and orodispersible films based on CEF-β-CD binary systems have been successfully realized to enhance the drug dissolution rate and mask its bitter taste [26,27].

On the contrary, no favorable effect on the stability of CEF aqueous suspensions was observed by β-CD addition and the authors attributed this result to the increased amount of drug in solution, due to the carrier solubilizing effect, coupled with the low complexing effect of β-CD towards the drug [28]. However, in all of the above cases, no evidence of the actual formation of the CEF-β-CD inclusion complex is given, and no data on the stability constant of the complex are provided.

On the other hand, it has been shown that the use of a third component can increase the CD–drug complexing power improving their effectiveness in use [24]. In fact, the addition of suitable auxiliary substances such as water-soluble polymers, organic acids and bases, or amino acids (AA) can have a synergistic effect in the drug solubility increase, allowing a reduction in the amount of CD required [29]. A strong stabilizing effect against CEF hydrolytic degradation was found by complexation with Captisol, alone or in the presence of the hydrophilic polymer Hypromellose, despite the very low stability constant of the complex (11.5 M^−1^) [30]. AA can be favorably employed for pharmaceutical applications, presenting different molecular structures, due to a large variety of functional groups, thus leading to different physicochemical properties. AA are generally recognized as safe excipients, also for pediatric patients, and their use as auxiliary substances for multicomponent complexation with CDs to improve the solubility and dissolution properties of drugs is having a growing interest [31,32,33,34,35]. In fact, AA can interact at the same time with both CDs, by hydrogen bond formation, and with drugs, by electrostatic interactions and salt formation. Jadhav et al. [36] evidenced a synergistic effect of Arginine in improving the solubilizing effect of βCD and hydroxypropyl-β-cyclodextrin (HPβCD) toward CEF.

Considering these findings, the aim of this work was the development of an oral solution of CEF suitable for pediatric use, exploiting complexation with CDs to improve drug solubility and taste masking in association with different AA. With this purpose, the complexation ability toward CEF of three different CDs, i.e., gamma CD (γ-CD) hydroxypropyl-β-cyclodextrin (HPβCD) and sulfobutylether-β-cyclodextrin (SBEβCD), selected as the safest CDs, being the only ones authorized also for parenteral use, was investigated. At the same time, the solubilizing effect of different kinds of AA on CEF solubility was also evaluated. Once selected the most effective CD and AA, the effect of their combined use, at different mol:mol ratios, on the drug solubility was investigated. The pH of all solutions was monitored, being another parameter to be checked for a well-tolerated pediatric oral solution. ^1^H-NMR spectroscopy was used to gain insight about the interaction in solution among the components in binary (drug-CD or drug-AA) or ternary (drug:AA:CD) systems. The best combinations were selected to prepare CEF solutions, in the presence or not of a suitable preservative, and subject to stability studies at three different temperatures. Finally, microbiological studies and odor assessment test were performed on the developed solutions in comparison with the commercial formulation to evaluate their antimicrobial activity and their actual ability to mask the unpleasant smell of the drug.

## 2. Materials and Methods

### 2.1. Materials

Cefixime trihydrate (CEF) was a gift from Menarini (Florence, Italy); hydroxypropyl-β-cyclodextrin (HPβCD, average molar substitution 0.62) was kindly donated by Roquette (Lestrem, France), sulfobutylether-β-cyclodextrin (SBEβCD, Captisol^®^, average substitution degree 6.8) by CyDex Pharmaceuticals Inc. (San Diego, CA, USA) and γ–cyclodextrin (γ-CD) from Cyclolab (Budapest, Hungary); l-Arginine (Arg), l-Histidine (His), l-Lysine (Lys), l-Glycine (Gly), l-Alanine (Ala), l-Proline (Pro), l-Serine (Ser), l-Leucine (Leu), l-Threonine (Thr), l-Glutamic Acid (Glu), l-Aspartic Acid (Asp) and sodium benzoate were provided from Merck KGaA (Darmstad, Germany). Purified water from Elix^®^ (Millipore, Burlington, MA, USA) and reagents of analytical grade were used.

### 2.2. Phase-Solubility Studies

Phase solubility studies were carried out in triplicate according to the Higuchi and Connors method [37] by adding an excess amount of CEF to 10 mL of purified water containing increasing concentrations of HPβCD or SBEβCD or γCD (0–25 mM) at a constant temperature of 25 ± 0.5 °C under magnetic stirring at 500 rpm up to equilibrium (72 h). Every 24 h an aliquot was withdrawn, filtered (0.45 µm pore size) and properly diluted for UV assay (Shimadzu UV/Vis 1601, Tokyo, Japan) at the drug λ_max_ of 287.2 nm. The analytical method was validated according to the ICH guidance Q2(R1) [38]. The linearity was assessed by a five-point calibration curve in the 4–20 mg/L concentration range. The linear regression equation was y = 0.0469x + 0.0072, with a correlation coefficient (R^2^) = 0.9995. Accuracy was determined by assaying three known concentration levels of the drug, corresponding to minimum, medium and maximum concentration values of the calibration curve, respectively. The precision of the analytical method was determined by using three drug concentration levels and three replications for each sample, to evaluate both repeatability and intermediate precision.

The LOQ and LOD value resulted 1.5 mg/L and 0.5 mg/L, respectively. No interferences due to the presence of CDs on the spectrophotometric measurements of CEF were noted (UV spectra of pure CD and His together with that of CEF are reported as Supplementary Material). The pH of suspensions was also constantly monitored using a Basic 20 pH-meter (Crison Instruments, Barcelona, Spain).

The apparent stability constant (K_1:1_) of the CEF-CD complexes was calculated from the slope of the phase-solubility diagrams and the CEF solubility in the medium in the absence of CDs (S_0_), according to Equation (1):

(1) $$\mathrm{Kc}=\frac{\mathrm{slope}}{S_{0}\times \left(1-\mathrm{slope}\right)}$$

The complexation efficiencies (CE) of CDs were also calculated by Equation (2) [39]:

(2) $$\mathrm{CE}=\frac{\mathrm{slope}}{\left(1-\mathrm{slope}\right)}$$

The solubilizing efficiency (SE) was determined by the ratio of drug solubility in the presence of the highest CD concentration used and that of the drug alone.

### 2.3. Solubility Studies

Solubility studies of CEF, alone or in binary mixtures with 11 different AA in the 1:1 and 1:2 molar ratios or in ternary 1:2:1 mol/mol CEF:aa:CD systems were performed in triplicate by adding an excess of the drug (alone or as binary or ternary system) to 10 mL of purified water. The suspensions were kept under magnetic stirring (500 rpm) at 25 ± 0.5 °C up to equilibrium (24 h). Aliquots of samples were withdrawn with a syringe-filter (pore size 0.45 µm), and suitably diluted for drug concentration assay at 287.2 nm (as described in Section 2.2). The absence of interferences due to other components was assessed. The pH of each sample was also measured.

#### H NMR Studies

All NMR spectra were recorded in D_2_O solution. The 2-D ROESY spectra were recorded by a Bruker Avance 400 spectrometer (400 MHz for ^1^H NMR) (Bruker Italia, Milan, Italy) at 300 K using a 5 mm probe and the pulse-sequence ROESYPH.2.

Two-dimensional ROESY spectra (rotating frame Owerhauser effect spectroscopy) were acquired with an acquisition time of 0.25 s, a spectral width of 10.2 ppm, a recycling delay of 2 s and a mixing time of 250 ms.

### 2.4. Molecular Dynamic (MD) Simulation

Molecular dynamics (MD) simulations of binary and ternary systems were performed using the GROMACS 5.0 package [40]. MD simulations were carried out in water under Periodic Boundary Conditions (PBC) to avoid problems with boundary effects caused by finite size, and make the system more like an infinite one, at the cost of possible periodicity effects. The visualization program DS ViewerPro 6.0 was used to build the initial systems conformations.

The partial atomic charge of the structures was calculated using the AM1-BCC method according to Pettersen et al. [41]. The OPLS All-Atom force field was applied to all structures [42]. All free molecules and complexes were solvated with water according to the Simple Point Charge (SPC) method [43]. In the simulation model, the molecules were placed in a cubic box of 4.1 × 4.1 × 4.1 nm^3^ with the sides of the box at a distance of about 10 Ǻ from the structures and solvated with 6800–7000 water molecules. The structures were relaxed by minimizing energy with the steepest descent algorithm (50.000 steps) and after two stage pre-equilibrations, the first one at constant Volume and Temperature (NVT) and the second at constant Pressure and Temperature (NPT), a 10 ns simulation was performed in the NPT ensembles at *T* = 300 K and *P* = 1 bar with a time step of 2 fs. The MD trajectories were registered every 0.1 ns. Long-range electrostatic interactions were calculated by the Ewald particle-mesh (PME) method [44], while the LINCS algorithm [42,43,44,45] was used to constrain the bond lengths.

The complexes were built “by hand” using the DS ViewerPro 6.0 program. Only the two most dissimilar conformations have been built: the guest has been introduced into the cavity in two symmetrically opposite ways. The task of exploring the permissible conformational space was left to the molecular dynamic simulation.

### 2.5. Preparation of Binary and Ternary CEF Liquid Formulations

Aqueous solutions of binary CEF:aa (1:2 mol/mol) or ternary CEF:aa:CD (1:2:1 mol/mol) containing 17 mg/mL of drug were prepared in the presence or absence of 0.5% *w*/*v* sodium benzoate as preservative.

### 2.6. Stability Studies during Storage

Stability studies from the chemical, physical and microbiological point of view of all the prepared solutions, in the presence or not of Na benzoate, were performed at 4 °C, 40 °C and room temperature up to 4 weeks. Chemical stability was weekly monitored by determining the residual drug concentration by spectrophotometric assay at λ = 287.2 nm (see Section 2.1). Stability was considered acceptable if drug concentration remained above 90% of the initial concentration. Physical and microbiological stability was weekly evaluated by visual inspection to check possible changes in organoleptic properties such as smell and color, precipitation or cloudiness phenomena, or mold formation, respectively. pH measurements were also weekly performed in order to detect possible modifications of the solutions (see Section 2.1).

### 2.7. Microbiological Studies

Microbiological tests were performed on *Escherichia coli* ATCC 35,218 grown in Luria Bertani (LB) broth.

#### 2.7.1. Minimal Inhibitory Concentration (MIC) Determination

Susceptibility tests were performed by a twofold standard broth microdilution method following the Clinical and Laboratory Standards Institute recommendations [46]. Overnight cultures of *E. coli* were diluted to reach OD_600nm_ values of 0.2; further 1:100 (*v*/*v*) dilutions were prepared to get inocula containing approximately 2 × 10^6^ colony forming units (CFU)/mL. More accurate back-calculation of viable titres of the inocula were performed by plate count method. Aliquots of 50 μL of inoculum were transferred in 96-well round bottom polystyrene Microtiter^®^ plates (Corning, NY, USA) containing 50 μL of preformed gradients of cefixime, cefixoral and binary and ternary oral formulations (stored at 4 °C and room temperature), to obtain approximately 1 × 10^5^ CFU/well in a final volume of 100 μL. For each antimicrobial, the minimum inhibitory concentration (MIC) value was detected in an Infinite M200 PRO Tecan microplate reader (Tecan^®^ France, SA-Lyon, France) by spectrophotometric determination of microbial growth at wavelength 600 nm (OD_600nm_) after 24 h incubation. MIC was the lowest concentration which prevents bacterial growth. Each assay was carried out at least in duplicate.

#### 2.7.2. Time Kill Test

Time-kill kinetics of Cefixoral, CEF-His and CEF-His-SBE ± CD solutions were evaluated in duplicate in LB broth against *E. coli* by the spot dilution method. Bacterial suspensions (approximately 1.0 × 10^6^ cell/mL) were incubated at 37 °C in the absence and presence of each sample at the corresponding MIC. Bacterial suspensions were serially diluted 1:10 and 5 μL aliquots of the dilutions were taken at 0, 1, 2, 3, 4 and 5 h and spotted on LB agar plates. After an incubation time of 24 h, single colonies in the spots were counted, and back-calculations are performed to determine the CFU/mL of the original sample. Values of CFU/mL showing a log_10_ reduction > 3, compared to control without antimicrobial, were interpreted as bactericidal activity, whereas 0 ≤ log_10_ reduction ≤ 3 was interpreted as bacteriostatic activity.

### 2.8. Odor Assessment Test

A group of ten (*N* = 10) healthy volunteers aged between 18 and 28 years (mean age = 22 years) were recruited as adult sensory panelists in a study aimed to evaluate the odor perception of CEF formulations. Each panelist was previously informed on what to do, i.e., test the odor of the solutions and all ones gave their consent Each participant was asked to fill a questionnaire according to the satisfaction rating, evaluated according to the ‘facial expressions scale’ or Facial Affective Scale, FAS [47] normally used for taste evaluation, just adapted to odor perception.

## 3. Results and Discussion

### 3.1. Phase-Solubility Studies

**Figure 2:**
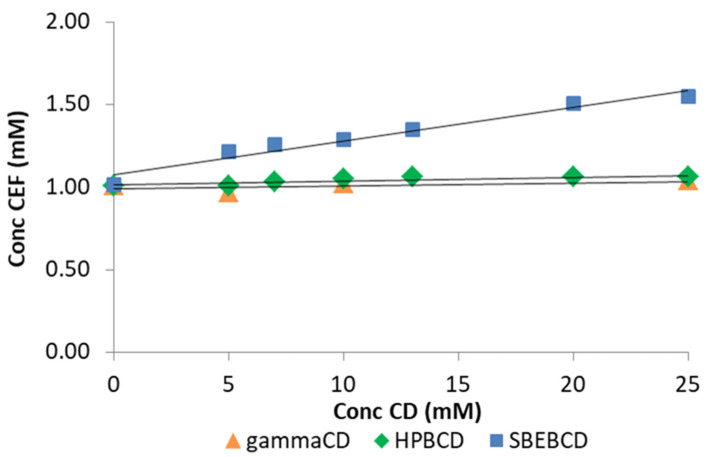
Phase-solubility diagrams of CEF in the presence of increasing concentrations (0–25 mM) of γCD (▲), HPβCD (♦) and SBEβCD (■) in water at 25 °C.

Figure 2 shows the phase-solubility diagrams of CEF in aqueous CDs solutions. CEF solubility slightly increased linearly only in the presence of increasing SBEβCD concentration, giving rise to an A_L_-type phase solubility diagram [37] with K_st_ ≈ 20 M^−1^, indicative of the formation of weak water-soluble complexes with possible 1:1 stoichiometry (Table 1). The low value of the K_st_, as well as the limited CEF solubility increase (about 1.5 times)_,_ were in perfect agreement with that reported in literature [30].

On the contrary, no drug solubility variations were observed in the presence of both γCD and HPβCD, indicating the absence of host-guest interactions, as confirmed also by the near to zero values of both Complexation Efficiency (CE) and Solubilizing Efficiency (SE) [48] (Table 1). To explain these findings, in the case of γCD it could be hypothesized that its cavity was unsuitable to effectively accommodate the CEF molecule. On the other hand, the unexpected result obtained when using HPβCD could be attributed to a steric effect of the hydroxypropyl substituents, which can hamper the drug access to the CD cavity [49].

On the other hand, in the case of SBEßCD, the steric hindrance due to the sulfobuthylether substituents was partly counterbalanced by electrostatic interactions between this polyanionic CD and the nitrogen-containing moieties of CEF, thus explaining its better complexing and solubilizing efficiency towards the drug [50].

The acidic pH of drug suspension (pH ≈ 3) remained almost constant also in the presence of each CD, up to the end of the experiment.

Thus, SBEβCD was selected for further studies, due to its, albeit weak, solubilizing power towards the drug, leading to a 1.5-fold increase of CEF aqueous solubility. The choice of this CD was further supported by literature data proving its wide, effective and safe use in various types of formulations, including parenteral and oral ones [50,51,52].

### 3.2. Solubility Studies

**Figure 3:**
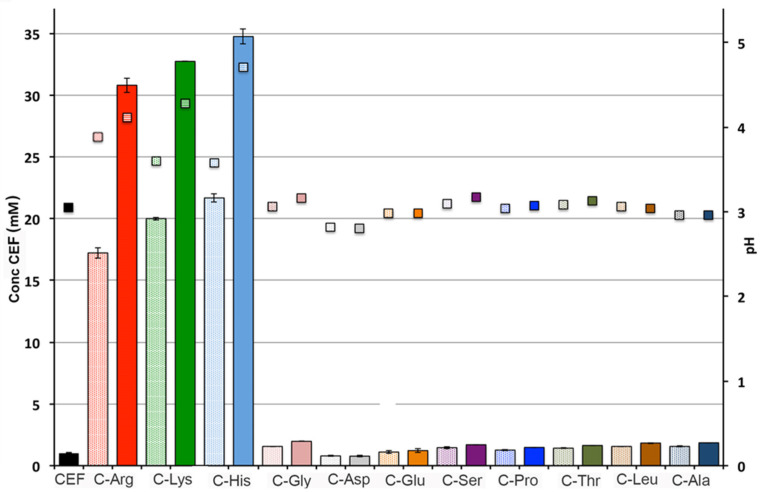
Aqueous solubility in water at 25 °C of CEF, alone or as a binary mixture with the different AA in the 1:1 (dotted bars) and 1:2 (full bars) molar ratios.

Considering the poor drug solubility improvement obtained by complexation with SBEβCD, the influence of different kinds of AA on CEF solubility was also investigated with the aim to develop an aqueous solution of CEF at a concentration as close as possible to that of the commercial suspension (20 mg/mL). Solubility studies of binary systems of CEF with AA in the 1:1 and 1:2 molar ratios were performed. AA were chosen based on their different properties in terms of basicity, acidity or polarity, in order to evaluate a possible different effect towards the drug due to their different nature. l-Arg, l-Lys and l-His were selected as basic AA; l-Gly, l-Ala, l-Pro and l-Leu as non-polar aliphatic AA, whereas l-Ser and l-Threo as polar ones. Finally, Asp and Glu were chosen as acid AA. As can be seen in Figure 3, among the examined AA, the basic ones were the most effective, in the order l-His > l-Lys > l-Arg, and their equimolar mixtures with the drug led to an about 22-, 20- and 17-fold increase, respectively, in CEF solubility. Their 1:2 binary mixtures allowed to increase drug solubility further significantly, achieving values higher than 30 mM. The best results were obtained in the presence of l-His, which enabled reaching a CEF saturation solubility of 35 mM, i.e., 35 times higher than the drug solubility and corresponding to 17.5 mg/mL, very close to the target value.

It should be highlighted that the achieved drug solubility value in the presence of His was much higher than that previously obtained by amorphous nanoparticles formation, where a maximum drug saturation solubility of 0.951 mg/mL was obtained [14] or about 3.5 mg/mL at 37 °C [15]. Moreover, the maximum CEF solubility increase obtained by the most effective co-crystal system (CEF:sodium acetate 1:1 mol:mol) was only about 18.5 times [16], i.e., about half of that obtained in the present work in the presence of His.

This finding can be reasonably ascribed to the acidic nature of the drug and the basic properties of such AA, and thus to a CEF salt formation, clearly more soluble than the drug as such. The improved solubility of various acidic drugs by the use of basic AAs as counterions has been already reported [32,53] A concomitant pH increase from the initial value of 3 (for the drug alone suspension) up to values of 4.1, 4.3 and 4.7 in the presence of l-Arg, l-Lys and l-His, respectively, was also pointed out.

On the other hand, the simple increase in pH (for example by NaOH addition and formation of CEF sodium salt) was not considered as a possible option, due to the low stability of CEF in basic solutions [30], which in fact until now prevented the development of aqueous solutions of this drug. No appreciable solubilizing effects were observed for all other examined AA, even when used at the 1:2 drug:AA mol/mol ratio.

**Figure 4:**
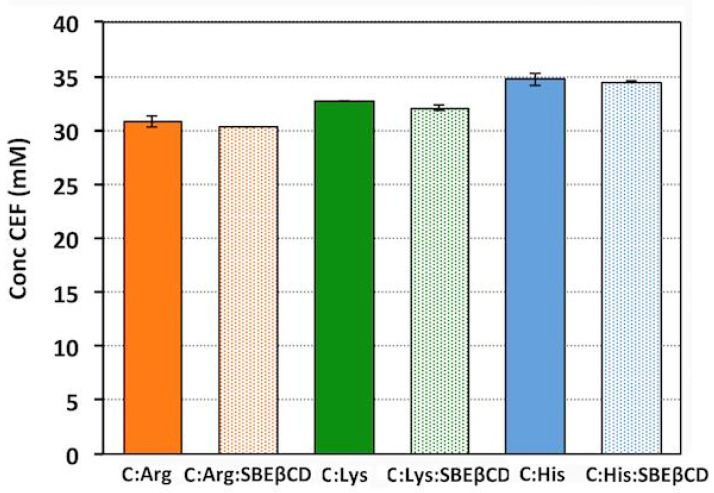
Solubility in water at 25 °C of CEF in binary (full bars) and ternary (dotted bars) mixtures with basic AA (Arg, Lys and His) and SBEβCD in the 1:2 and 1:2:1 molar ratios.

Based on these results, solubility studies of ternary mixtures CEF:basic AA:SBEβCD (1:2:1 mol/mol) were then carried out, in order to evaluate if their combined use could have a synergistic effect on CEF solubility enhancement. In fact, it has been shown that the addition of suitable auxiliary substances can improve the cyclodextrin solubilizing and complexing properties due to the formation of a multicomponent complex [54,55]. Unlike that expected, CEF solubility in ternary mixtures was very similar to that obtained in the presence of the basic AA alone, showing no additional effects due to the joined presence of CD and resulting even lower than the theoretical one calculated as the sum of CEF solubility in the presence of SBEβCD and amino acid separately (Figure 4).

It was not considered necessary to perform X-Ray Powder Diffraction studies on the end solids from solubility studies, in order to obtain data about possible CEF solid state modifications that occurred during the text, which could be responsible for the observed changes in its saturation solubility. In fact, a possible increase in drug solubility as a consequence of some solid-state change, due to the formation of a more soluble hydrate or polymorphic form, or to its partial amorphization can be excluded on the basis of the obtained results. Indeed, the CEF solubility increase was obtained only in the presence of the basic AA, and it was reasonably attributed, as above discussed, to the CEF salt formation. In addition, no further increase in drug solubility was observed in the ternary CEF:basic AA:CD system, despite the presence of the amorphous SBEβCD, thus allowing to exclude also in this case any contribute of possible changes in drug solid state properties to its solubility increase.

A possible competitive effect of basic AA with the drug in the interaction with the CD can be hypothesized. In fact, cyclodextrin complexation with AA has been reported [55,56]. However, interestingly, the pH of the solutions containing the ternary system was higher than that of the binary CEF:His solution (5.6 vs. 4.7) resulting in a more tolerable pediatric oral liquid formulation.

Based on such results, His was selected among basic AA due to not only its highest solubilizing power towards the drug, as emerged by solubility studies, but also to its essential role in the protein formation, whose deficiency can lead to growth problems in children [57,58].

The use of SBEβCD was also investigated, to evaluate any possible stabilizing and taste-masking effects, in addition to its observed positive role in enhancing the solution pH.

In fact, the stabilizing effect of SBEβCD against hydrolytic degradation of CEF [30], as well as the very successful use of SBEβCD as a taste-masking agent in pediatric oral formulations [59] have been previously reported.

### 3.3. Molecular Dynamic (MD) Simulation

MD simulations were performed to get more insight about the possible interactions between CEF, His and SBEβCD molecules. In MD simulations, CEF and His were introduced from both sides of the SBEβCD cavity, in two symmetrically opposite ways. In all the simulations SBEβCD was considered in anionic form (SBEβCD^7−^ total charge 7), CEF was considered both in neutral (CEF^0^) and in anionic form (CEF^2−^) and His as neutral (HIS^0^), zwitterionic (HIS^zw^) or protonated zwitterionic form (HIS^zw1+^). The stability of the CD complexes was evaluated by the gain of potential energy of the complex (EP_gain_) compared to the sum of the potential energy of the free components, according to Equation (3):

(3) $${\mathrm{EP}}_{\mathrm{gain}}={\mathrm{EP}}_{\mathrm{complex}}-\left({\mathrm{EP}}_{\mathrm{host}}+{\;\mathrm{EP}}_{\mathrm{guest}}\right)$$

The potential energy is reported as the average of the potential energy calculated for each conformation recorded during the MD simulation. The conformations collected were 100.

The MD simulation results are collected in Table 2.

For each pair of conformers, the most favorable EP_gain_ has been reported. As can be observed the complex formed by SBEβCD^7−^ and the ionized form of CEF (CEF^2−^) showed an EPgain not consistent with its actual existence (238 KJ/mol), while the complex between CD and neutral CEF (SBEβCD^7−^-CEF0) would be quite stable, achieving an EPgain of −313 KJ/mol. From the NMR spectra (see below) the SBEβCD-CEF complex does not seem to exist in detectable quantities, therefore it is reasonable to assume that CEF is mainly present in the deprotonated form. Such a result could be attributed to the lower affinity of the ionized form of the drug for the inclusion into the apolar CD cavity, due to its less hydrophobic properties.

A strong interaction, probably of electrostatic nature, occurred also between SBEβCD^7−^ and the His protonated zwitterionic form (HIS^zw1+^) as demonstrated by the corresponding EP_gain_ of −301 KJ/mol. On the other hand, His strongly interacted, by salt formation, also with CEF, when they were both in the charged form, as HIS^zw1+^ and CEF^2−^ The EP_gain_ of their interaction was of the same order of magnitude of that related to the SBEβCD^7−^- HIS^zw1+^ interaction (−317 KJ/mol vs. −301 KJ/mol). This result seems to confirm the hypothesized competition between CEF and His for interacting with SBEβCD, resulting in absence of synergism between SBEβCD and His in drug solubilization.

### 3.4. H NMR

**Figure 5:**
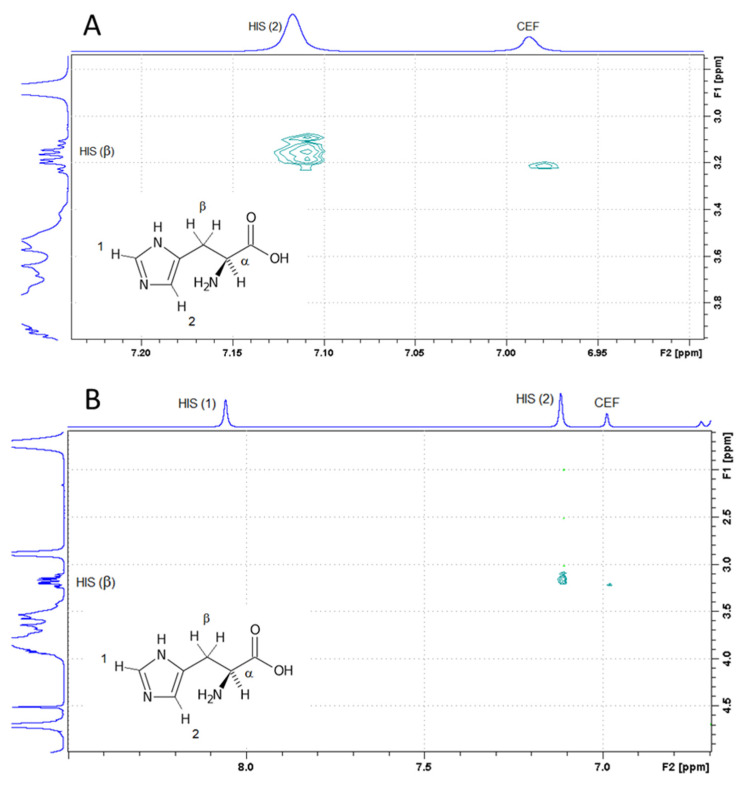

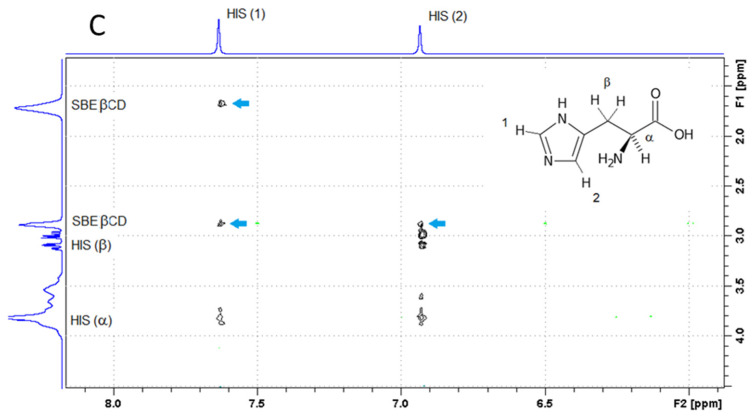
2D ROESY spectra of ternary CEF:His:SBEβCD (**A**,**B**) and binary His:SBEβCD (**C**) solutions.

2-D ROESY experiments were performed in order to gain further insight into the possible interactions between the components in binary and ternary systems. Figure 5A,B shows the spectra related to the ternary CEF:His:SBEβCD system, where intermolecular cross peaks, due to nuclear Overhauser effect (nOe) between the protons of CEF and the α and β protons of His were observed, thus demonstrating the interactions between the two molecules. On the contrary, no interactions between His and SBEβCD or CEF and SBEβCD were detected in the ROESY spectrum of the ternary system. An interaction was instead observed in the ROESY spectrum of the solution containing only SBEβCD and His, as proved by a nOe signal between the protons of the amino acid heterocycle (1 and 2) and the butyl protons of SBEβCD (Figure 5C), thus confirming the MD findings.

Therefore, from these results it can be concluded that, when all three components are simultaneously present in solution, only salt formation of CEF with the basic His occurs, without any complex formation between CEF^2−^ and SBEβCD^7−^ or detectable interaction between HIS^zw1+^ and SBEβCD^7-^. In fact, in such conditions, CEF is almost totally in its ionized form, and thus unable to interact with the CD, as shown by MD experiments, while in the case of the basic amino acid, evidently, the interaction with the acidic drug is prevalent with respect to that with the anionic CD.

### 3.5. Preparations of Oral Liquid CEF Formulations

According to the results obtained from solubility studies, four different aqueous solutions of CEF at 17 mg/mL were prepared, containing the drug as a binary system with His or as ternary system CEF:His:SBEβCD at the 1:2 and 1:2:1 molar ratios, respectively, in the presence or not of Sodium Benzoate (0.5% *w*/*v*) as a preservative, whose safe use for oral daily administration in pediatric patients has been demonstrated by Embrechts et al. [60].

### 3.6. Stability Studies of CEF Solutions

**Figure 6:**
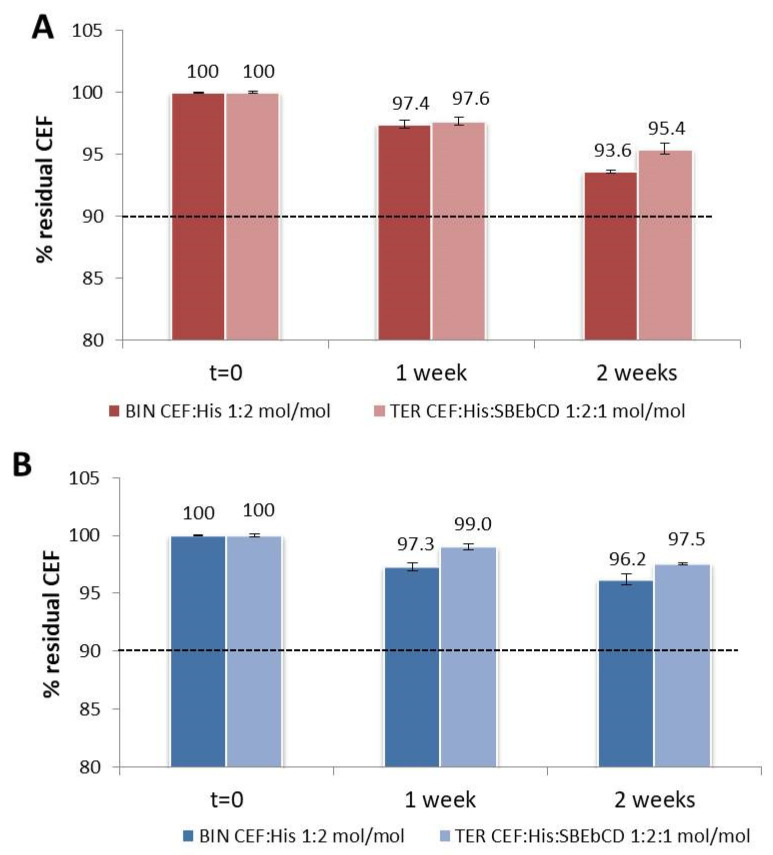
Stability studies on the binary CEF:His and CEF:His:SBEβCD ternary solutions stored at room temperature (**A**) or at 4 °C (**B**) in light-protected glass vials.

Stability studies were performed on the developed CEF solutions stored at room temperature, at 40 °C and at 4 °C, protected from light, by weekly monitoring the drug concentration, possible pH variations, organoleptic properties modifications or mold formation. Samples stored at 40 °C showed a marked color change from colorless to orange just within the first week of storage, together with a drastic reduction in CEF concentration up to about 75% of the initial value (data not shown). Figure 6 shows the results expressed as residual CEF% of samples stored at room T (Figure 6A) and 4 °C (Figure 6B) without Na Benzoate. Similar results were obtained for the corresponding preserved samples, thus indicating no effects exerted by the preservative Na benzoate on samples’ stability.

All the prepared formulations remained stable during the two weeks in terms of residual drug%: in fact, it was in all cases higher than 90%, which is the limit of drug labeled potency currently accepted for pharmaceutical dosage forms. Storage at 4 °C slightly, but not significantly (*p* > 0.05), delayed the CEF degradation rate if compared to room temperature. Moreover, ternary systems-based formulations always showed % residual concentration values a little, even if not significantly (*p* > 0.05) higher than the corresponding binary system-formulations, thus indicating a certain stabilizing power of SBEβCD. In fact, the ternary system formulation stored at 4 °C was the only one whose residual drug content was not significantly different from *t* = 0 after two weeks of storage.

The pH of all the solutions did not change during storage, remaining at values of 4.7 and 5.6 for the binary and ternary formulations, respectively, only registering a 0.1 pH unit change during the second week of storage for both the solutions stored at room temperature.

Different behavior for the color change of the solutions was observed during the second storage week between formulations stored at room temperature and in refrigerated conditions. The latter started to change from transparent to pale yellow whereas the first ones became a bright yellow color.

Thus, it seemed to be worthy of interest to investigate if the color variation could be related to a loss of the drug activity. Controversial opinions are reported in the literature concerning the reason for such a color modification. Some authors state that the presence of sulfur can play an important role in the yellowing process, whereas other authors attribute the change in color to the beta-lactam ring hydrolysis, thus leading to loss of activity [61,62].

### 3.7. Antimicrobial Activity

To evaluate the actual maintenance of antibacterial activity of CEF in the selected drug formulations, the MICs of CEF-His (1:2 mol/mol) and CEF-His-SBEβCD (1:2:1 mol/mol) systems were measured after solution reconstitution and up to 14 days (spanning the usual therapy period). MICs of binary and ternary systems were compared to those of fresh suspensions of commercial Cefixoral^®^ formulation (CEF 100 mg/5 mL) and of pure CEF. A binary CEF-SBEβCD (1:1 mol/mol) sample was also tested to investigate the role of CD alone on drug stability.

**Figure 7:**
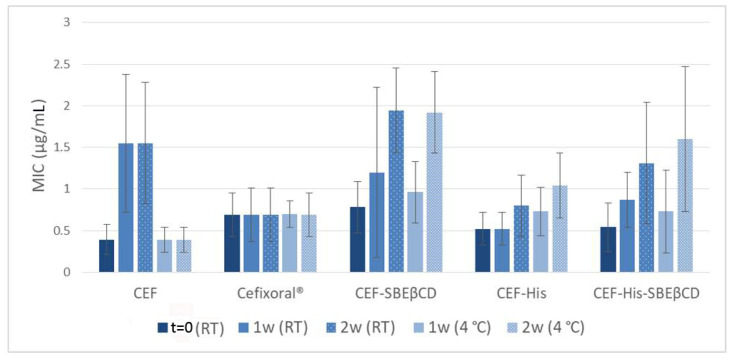
Minimum Inhibitory Concentration (MIC) values of CEF and its different formulations against *E. coli* at *t*_0_ and after 1 and 2 weeks of storage at room temperature (RT) or at 4 °C.

The results of these studies showed a trend towards a slight increase of MIC values over time, indicative of some loss of efficacy, for all samples, with the only exception of Cefixoral^®^ suspension (Figure 7). Furthermore, no benefits were observed for samples stored under refrigerated conditions (4 °C) with respect to those kept at room temperature. The greater stability of the antibacterial activity of Cefixoral^®^ could be attributed to the gradual dissolving of the drug in the commercial suspension; as known, only the soluble form of CEF is sensitive to hydrolytic degradation, while not yet solubilized drug is preserved. Among the binary and ternary CEF systems evaluated, CEF-SBEβCD always showed the highest MIC values. However, the observed increases of MIC were non-statistically significant (*p* > 0.05). The lack of statistical significance should be mainly attributed to the inherently experimental variability of the method used; in fact, it is generally accepted that broth MIC tests are reproducible to within one doubling dilution of the real end point (i.e., ±one well or tube in a dilution series) [63]. Overall, the binary and ternary CEF systems have been shown to maintain their antibiotic activity over 14 days, that is the time of the intended use in therapy.

**Figure 8:**
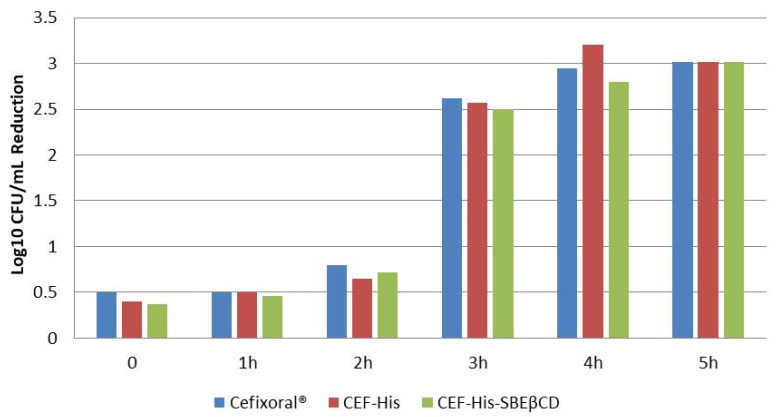
Time kill assay for the evaluation of antimicrobial effectiveness of Cefixoral^®^ suspension, and CEF-His 1:2 mol/mol and, CEF-His-SBEβCD 1:2:1 mol/mol solutions against *E. coli* expressed as Log_10_ CFU/mL reduction at different times.

The results of the time-kill kinetics showed that both Cefixoral^®^ suspension and binary (CEF-His) and ternary (CEF-His-SBEβCD) solutions gave rise to similar killing kinetics that reach a complete killing (Log10 CFU/mL reduction > 3) after 5 h (Figure 8), confirming the bactericidal activity of CEF in *E. coli* [63].

Microbiological studies enabled to support the results of stability studies and the conclusion that the yellowing of the binary and ternary CEF solutions, observed during the storage, was not correlated to a significant loss of antimicrobial activity. Further reinforcing this conclusion is the observation that yellowing over time was also observed in the Cefixoral^®^ suspension, despite its MIC values remaining almost constant during the whole storage period considered.

### 3.8. Adult Sensory Panel Test

An unpleasant taste or odor can hinder children’s acceptance of medicine. Authorities recommend palatability assessments involving children whenever possible but when not possible adult panels for taste-screening can provide an alternative method to be examined for result transferability to children [64].

A group of ten healthy adult volunteers was recruited as adult sensory panelists in the evaluation of the odor perception of the binary and ternary solutions.

Each volunteer was asked to evaluate its odor perception using a rating scale called ‘facial expression scale’ or ‘Facial Affective Scale’ (FAS), typically used for taste evaluation in children, just adapted for the odor evaluation of the formulations. The FAS is designed to capture the emotional (affective) reaction of panelists and includes both happy and sad facial expressions, ranging from the happiest feeling possible to the saddest feeling possible, depicting decreasing gradations of acceptability. All subjects showed their strongest dislike for the binary CEF:His solution, judged to have a really bad smell. On the contrary, no sensation was revealed from volunteers for the ternary CEF:His: SBEβCD solution, which was found to be odorless (Table 3).

These results proved that the presence of SBEβCD was successful as an unpleasant-odor-masking agent. This could appear an unexpected effect, considering the low stability constant of its complex with CEF. However, it should be taken into account that the olfactory perception threshold, especially in the case of sulphurated compounds, is extremely low, in the order of ppb. Therefore, even if the fraction of complexed drug molecules is limited, it was probably enough to bring down the concentration of free drug molecules below the olfactory perception threshold.

Moreover, an effective masking effect of the bitter taste of CEF has been also obtained in the presence of βCD [26] despite its even lower complexing power towards the drug, has emerged in our preliminary phase-solubility studies (data not shown). Evidently, besides drug complexation, other mechanisms can play a role in the masking effect of CDs against unpleasant odors or tastes [65].

The obtained masking effect of the unpleasant drug organoleptic properties represents an actual advantage for its oral administration, particularly in pediatrics, allowing to improve children’s compliance.

## 4. Conclusions

Preliminary phase-solubility and solubility studies enabled the selection of SBEβCD and His as the most effective CD and amino acid, respectively, to be used in combination for the development of a CEF oral solution suitable for pediatric use.

In fact, His showed the highest solubilizing power towards the drug compared to all tested AA (including the basic ones Arg and Lys), allowing to increase CEF solubility from 0.76 mg/mL up to 17.5 mg/mL, very near to the concentration of the commercial CEF suspension (100 mg/5 mL), thus eliminating the possible problems of poor dosage accuracy, in virtue of its complete homogeneity, and of unpleasant texture sensation in the mouth of pediatric patients.

On the other hand, the presence of SBEβCD, while not giving rise to a further improvement in CEF solubility, was essential in improving the formulation performance. In fact, the ternary CEF-His-SBEβCD solution resulted superior to the binary CEF-His one in terms of better tolerability, due to its higher pH (5.6 vs. 4.7) and better acceptability by pediatric patients, due to the ability of SBEβCD to completely mask the unpleasant drug odor, as assessed by a sensory panel test. This resulted in improved patient compliance, which is one of the most important issues influencing the adherence to the therapy and its effectiveness, especially in the case of pediatric patients.

MD and NMR studies were useful to gain further insight about the possible interactions between the components in binary and ternary systems. Interactions between the CEF and His were observed, while no interactions between His and SBEβCD or CEF and SBEβCD were detected for the ternary system. An interaction was instead observed for the solution containing only SBEβCD and His.

Moreover, stability and microbiological studies proved that the developed solution was stable during the common two-weeks-use in therapy, without the need of adding any preservative, without the need of adding any preservative or other excipients potentially harmful for pediatric patients and maintained almost unchanged the drug antimicrobial activity.

Thus, the developed CEF-His-SBEβCD solution can be proposed as a valid alternative to the commercial CEF oral suspension, avoiding problems of unpleasant texture and taste and thus improving patient compliance, and also allowing an easier administration and a more accurate dosing and dose-adaptability, in virtue of its complete homogeneity.

## Figures and Tables

**Table 1:** Stability constant (K_st_), complexation efficiency (CE) and solubilizing efficiency (SE) of CEF complexes with γCD, HPβCD or SBEβCD in water at 25 °C.

	K_st_ (M^−1^)	CE	SE
γCD	1.8	0.002	1.03
HPβCD	2.3	0.002	1.04
SBEβCD	19.7	0.021	1.53

**Table 2:** Potential Energy gain (EP_gain_) of the different binary systems CEF:SBEβCD and CEF:His calculated by MD simulation.

Complex	EP_gain_ (KJ/mol)
SBEβCD^7−^-CEF^0^	−313
SBEβCD^7−^-CEF^2^	−238
SBEβCD^7−^-HIS^0^	−101
SBEβCD^7−^-HIS^zw^	−161
SBEβCD^7−^-HIS^zw1+^	−301
2HIS^0^-CEF^0^	−43
2HIS^zw^-CEF^0^	−125
2HIS^zw^-CEF^2−^	−163
2HIS^zw1+^-CEF^2^	−317

**Table 3:** ‘Facial Affective Scale’ (FAS) rating for the odor evaluation carried out by 10 healthy volunteers.

Odor Perception		Binary Solution CEF:His	Ternary Solution CEF:His:SBE CD
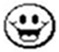	Extremely good odor	0/10	0/10
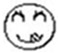	Very good odor	0/10	0/10
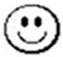	Pleasant odor	0/10	0/10
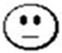	Odorless	0/10	10/10
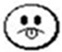	Unpleasant odor	0/10	0/10
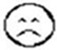	Very bad odor	0/10	0/10
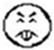	Extremely bad odor	10/10	0/10

## Data Availability

Not applicable.

## Supplementary Materials

The following are available online at https://www.mdpi.com/article/10.3390/pharmaceutics13111923/s1, Figure S1: UV spectra.
