# Supplementary material for: Combined Use of Cyclodextrins and Amino Acids for the Development of Cefixime Oral Solutions for Pediatric Use
Source: Pharmaceutics. 2021 Nov 13;13(11):1923. doi: 10.3390/pharmaceutics13111923 (PMC8620304; doi:10.3390/pharmaceutics13111923)
Supplement: Supplementary file 1 [file pharmaceutics-13-01923-s001.zip › pharmaceutics-1423785-supplementary.pdf]

pharmaceutics

Supplementary Materials: Combined Use of Cyclodextrins and Amino Acids for the Development of Cefixime Oral Solutions for Pediatric Use

Marzia Cirri, Natascia Mennini, Giulia Nerli, Jessica Rubia, Enrico Casalone, Fabrizio Melani, Francesca Maestrelli and Paola Mura

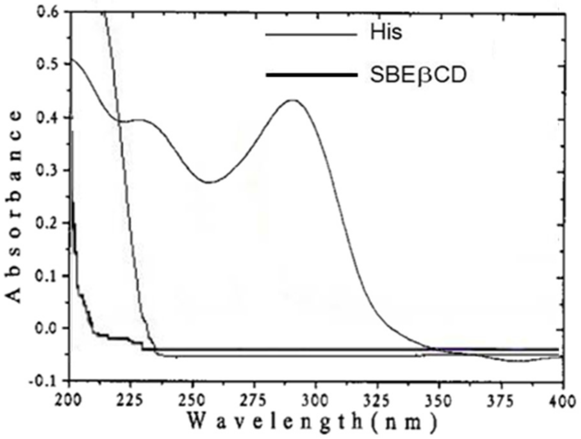

Figure S1. UV spectrum of pure CEF (λ_{max} = 287.2 nm) together with those of SBEβCD and His.
